# Medical Charities in Victoria

**Published:** 1904-07-30

**Authors:** 


### MEDICAL CHARITIES IN VICTORIA.

The Easter season saw a large bazaar carried through successfully by the Order of the Druids in aid of the funds of the Melbourne Hospital. The result is expected to be from £1,000 to £1,500. "The Melbourne," as our largest general hospital, receives cases of every kind from every corner of the State. Even maternity cases sometimes find their way there, though for obvious reasons they are not retained, but as soon as possible are sent to the Women's Hospital, distant about fifteen minutes' drive. The support is not as general as the supply of patients; very little money is received from distant parts of the State, and in Melbourne itself contributions are much more limited than they ought to be. As the readers of The Hospital are aware, there is no poor rate anywhere in the Australian Commonwealth, though such a rate has been proposed. Its place is partly taken by the Government grant which used to be pound for pound of private contributions, but has been much reduced of late years everywhere. A proposal which meets with approval in many quarters has been frequently urged lately—that is, that admission moneys to racing and other clubs which occupy lands received from the Government, and also the admission moneys at all outdoor sports and the value of the prizes offered at such sports, be taxed 5 per cent., the money so received to go towards the support of the charities. It is improbable that anything definite will be done on these lines, for our people are generous. As times improve, money will flow again towards the charities, and taxes and other modes of permanently supporting the hospitals will be postponed until another day.

The Government grant to the Melbourne Hospital was reduced by £2,000 in 1893–4, and again in 1902–3 by £2,160, leaving it with £9,840. This necessitated a special appeal, which was so far successful that the closing of some of the large wards, as had been proposed, was obviated. The prospects for this year are very slightly better; the hospital is always overcrowded, and the committee and secretary are in a state of constant anxiety. For the year ending June 30th, 1903, the deficiency of income was £4,000, the total expenditure being £26,588.

In spite of the difficulties of the position—a hospital building out of date and constant monetary deficiencies—an immense amount of good work is done in the Melbourne Hospital. Although the lack of time-saving and labour-saving appliances must fall heavily on the staff, the medical cleanliness of the hospital and the efficiency of the nursing methods compare well with more favoured institutions. The average daily number of in-patients is 292·5. This obviously presents a wide and excellent field for the medical student; and the Melbourne rightly takes rank not only as the leading general hospital—it is the largest—but also as the primary medical training school of the State. Accommodation for medical students, in the way of lecture and post-mortem rooms and laboratories, has been extended and entirely remodelled during the past three or four years. A large room is now thoroughly equipped for X-rays practice, and in many ways and under many disadvantages the committee endeavours to keep the institution up to date with the latest discoveries.

The fever hospital, built two years ago, has not yet been opened, on account, it is alleged, of the reluctance of some of the municipalities of the State to co-operate with the Government in its maintenance. Meantime the Melbourne has to receive all the cases that ought to go to a fever hospital, including diphtheria. The past two summers have been unusually cool and wet, and both have seen an increased number of typhoid cases, generally of a more severe type than usual. This should be an interesting fact to the medical experimenter. The average Australian lay mind has hitherto supposed that the hotter the summer the more numerous and severe the typhoid.

Victoria is still without provision for epileptics other than that afforded by the asylums for the insane. This defect is likely to be remedied ere another year has passed. The matter has been taken up by the National Council of Women, which counts amongst its delegates three of the women doctors of Melbourne, Drs. Jean Greig, Constance Ellis, and Mary Page Stone. The Council appointed a sub-committee; and calling to their assistance experts like Dr. Fishbourne, ex-physician to the Lunacy department, and Dr. Springthorpe, visiting physician to the asylums, the members have drawn up a scheme for a colony on the plan followed in England and in Germany, a colony that should ultimately become self-supporting. Nothing but the want of funds stands in the way of the realisation of the project.

Our Board of Health are busy taking precautionary measures to keep out the bubonic plague, which has again reared its head in two of the neighbouring states, New South Wales and Queensland.

[5] For particulars respecting hotels, boarding-houses, apartments, consult publications of G. W. and L. S. W. Railways; "C. T. C. Handbook and Guide." London: 47 Victoria Street, S.W. 1904. "Holiday Resorts and Recommended Addresses." London: The Teachers' Guild, 74 Gower Street, W.C. 1904.

